# Exogenous Melatonin Application Delays Senescence of Kiwifruit Leaves by Regulating the Antioxidant Capacity and Biosynthesis of Flavonoids

**DOI:** 10.3389/fpls.2018.00426

**Published:** 2018-04-05

**Authors:** Dong Liang, Yanqiu Shen, Zhiyou Ni, Qin Wang, Zhi Lei, Nengqin Xu, Qunxian Deng, Lijin Lin, Jin Wang, Xiulan Lv, Hui Xia

**Affiliations:** ^1^College of Horticulture, Sichuan Agricultural University, Chengdu, China; ^2^Institute of Pomology and Olericulture, Sichuan Agricultural University, Chengdu, China

**Keywords:** melatonin, kiwifruit, natural senescence, flavonoid biosynthesis, antioxidant capacity

## Abstract

Melatonin, a multiple signal molecule, plays important roles in delaying senescence during the development of plants. Because few species have been studied for the effect of exogenous melatonin on anti-aging, the plausible mechanism of melatonin of anti-aging effects on other plant species has remained largely unknown. In the present study, the effects of exogenous melatonin on leaf senescence in kiwifruit were examined during natural aging after melatonin (200 μM) or water (Control) pretreatment. The decreased membrane damage and lower hydrogen peroxide (H_2_O_2_) content due to the enhanced scavenging activity of antioxidant enzymes peroxidase (POD), superoxide dismutase (SOD), and catalase (CAT) demonstrated that melatonin effectively delayed the aging of kiwifruit leaves. Likewise, owing to up-regulated expression of chlorophyll a/b-binding protein (*CAB*) gene in the sampled leaves pretreated with melatonin, chlorophyll degradation decreased. Therefore, osmoregulatory substances in sampled leaves accumulated (e.g., soluble sugar and soluble protein) and seedling cell environment stability was maintained. Simultaneously, melatonin decreased H_2_O_2_ concentration owing to increased glutathione (GSH) and ascorbate (AsA) content, and the expression levels of glutathione reductase (*GR*), ascorbate peroxidase (*APX*), monodehydroascorbate reductase (*MDAR*), dehydroascorbate reductase (*DHAR*) were up-regulated by melatonin application, indicating that the increase of GSH and AsA was attributed to the expression of these genes. In addition, a large amount of flavonoids accumulated in seedlings pretreated with melatonin, and transcript levels of eight genes involved in flavonoid synthesis, including phenylalanine ammonia-lyase (*PAL*), cinnamate-4-hydroxymate (*C4H*), chalcone synthase (*CHS*), flavanone 3-hydroxylase (*F3H*), flavonol synthase (*FNS*), leucoanthocyanin reductase (*LAR*), anthocyanin reductase (*ANR*), flavonoid 3-*O*-glucosyltransferase (*UFGT*) were enhanced in response to melatonin application. These results indicated that melatonin delayed aging of kiwifruit leaves by activating the antioxidant capacity and enhancing flavonoid biosynthesis. All of these results can provide clear proof that melatonin plays a key roles in delaying leaf senescence.

## Introduction

Senescence, the final stage in the leaf development, is programmed and complex process, which is regulated by developmental and environmental factors. Cells in those tissues experience several drastic changes in metabolism (Quirino et al., 2000; Lim and Nam, 2007; Kim et al., 2017). These changes include degradation of chlorophyll and active macromolecules, recycling of nutrients, transcriptional level of senescence-associated genes, and accumulation of excess of harmful free radicals (Inada et al., 1998; Thompson et al., 1998). Although a crucial and evolutionarily physiological process for plant fitness, senescence of plant leaves affect plant biomass accumulation and flower bud differentiation, and yield in future, resulting in economic losses. Therefore, an improved understanding of leaf senescence must be explored, and novel strategies for preventing or delaying this process in certain environment must be developed to enhance ecological and safety of plant.

Melatonin (*N*-acetyl-5-methoxytryptamine), an indole molecule, was extracted in 1958 from the bovine pineal gland (Lerner et al., 1958, 1959). Since the discovery of melatonin, much progress has been made in unraveling its role in plants. Previous studies speculated that mitochondria and chloroplasts are the original synthesis sites of melatonin according to melatonin’s primary function and evolution in eukaryotes. Mitochondria and chloroplasts are major producer of free radical. High levels of melatonin in mitochondria and chloroplasts are used to protect these important cellular organelles against oxidative stress and preserve their physiological functions. (Tan et al., 2013; Manchester et al., 2015; Reiter et al., 2017).

A primary function of melatonin in plants is to act as an antioxidant. Melatonin works in plant by decreasing ROS, reducing menbrane lipid peroxidation and up-regulating of antioxidant enzymes activity (glutathione peroxidase, superoxide dismutases, and catalase et at.) (Tan et al., 2002; Rodriguez et al., 2004; Reiter et al., 2014). It can act as a growth regulator in a similar way as the auxin, indole-3-acetic acid (IAA) does, governing the growth of roots, shoots, and explants (Murch et al., 2001; Hernandez-Ruiz et al., 2004, 2005; Arnao and Hernández-Ruiz, 2014). Chen et al. (2009) detected 0.1 μM melatonin stimulated the roots growth of young wild leaf mustard seedlings (*Brassica juncea*), while 100 μM inhibited. Furthermore, exogenous supplement of 0.1 μM melatonin improved the endogenous levels of free IAA in roots of young seedlings. In addition, melatonin can delay senescence as a biological stimulant, which was demonstrated by previous studies (Tal et al., 2011; Zhang et al., 2013; Wang et al., 2014). Moreover, melatonin protects plants against a variety of environmental stresses, such as cold (Ding et al., 2017), drought (Cui et al., 2017), salinity (Arnao and Hernandez-Ruiz, 2009; Wang et al., 2016), and heavy metal toxicity (Gu et al., 2017; Nawaz et al., 2018). A study utilized mRNA-seq technology to analyze the effect of melatonin on genome-wide gene expression, and monitored a large number of differentially expressed genes: (i) genes involved in plant stress defense: many stress receptors, kinases, and stress-associated calcium signals were up-regulated, chlorophyllase and PaO, involved in chlorophyll degradation, were both down-regulated; in addition, cell death associated genes were mostly down-regulated, (ii) genes involved in hormone signaling: most identified genes in ABA, ethylene, salicylic acid, and jasmonic acid pathways were up-regulated, while genes associated to auxin responses, homeostasis, signaling, peroxidases, and those associated with cell wall synthesis and modifications were mostly down-regulated (Weeda et al., 2014). The interesting results may indicate the reason why melatonin possesses antioxidant ability and the role of melatonin in stress defense and delaying senescence.

Excess and uncontrolled accumulation of reactive oxygen species (ROS) is responsible for the onset of senescence. ROS levels and their destructive effects are known as the common action the plant response to senescence (Apel and Hirt, 2004; Khanna-Chopra, 2012). Many researchers have demonstrated that melatonin can protect organisms against ROS (Tan et al., 2007; Li et al., 2012; Wang P. et al., 2012; Shi et al., 2015; Zhang et al., 2016; Gong et al., 2017). Its antioxidant activity appears to function via the following pathways: (i) scavenging free radicals directly, (ii) stimulating antioxidant enzymes, such as peroxidase (POD), superoxide dismutase (SOD), and catalase (CAT), (iii) increasing the activities of other antioxidants, e.g., ascorbic acid (AsA), soluble sugars, and flavonoids, (iv) protecting antioxidant enzymes from oxidative damage, and (v) enhancing the efficiency of the mitochondrial electron transport chain, thereby easing electron leakage and, to reduce the content of free radicals (Li et al., 2012; Wang P. et al., 2012; Shi et al., 2015; Zhang et al., 2016; Zhou et al., 2016; Gong et al., 2017). However, regulation mechanisms involved in the senescence of leaves by melatonin is rarely reported in plants, particularly fruit trees.

The objective of the present study was to investigate the effects of melatonin in delaying senescence and to analyze the mechanisms induced by melatonin. Here, we were able to detect the changes in physiology and molecules, included chlorophyll concentrations, membrane lipid peroxidation degree, H_2_O_2_ content, et al. Specifically, we analyzed the content of GSH, AsA, and phenolic compounds, and studied the gene expression of related enzyme in the AsA-GSH cycle and the flavonoid biosynthetic pathway to highlight the exact mechanism of melatonin-induced antioxidant properties.

## Materials and Methods

### Sample Collection, Plant Material Preparation, and Melatonin Application

In the present study, kiwifruit seedlings were used as study materials. The plant seeds were collected in September 2016. We soaked the seeds in 800 mg⋅L^-1^ gibberellin solution for a day and stratified them at 4°C for 2 months. For inducing germination, the seeds were placed in incubator at 25°C for 8 h and at 4°C for 16 h for 2 weeks. Thereafter, the seeds were incubated at 25°C until sprouting occurred. The germinated seeds were planted in a seedling tray and covered with 2 mm nutrient soil layer. They were cultured in an artificial climate room (light/dark cycle: 12 h/12 h, temperature: 25°C/20°C). When the seedlings grew to have two to three true leaves, the seedlings with similar growth were transferred into plastic pots (20 cm in diameter) containing perlite and placed in a greenhouse before treatment. All the lateral branches were carefully removed.

After 4 months of growth in 2017, healthy and uniform plants were assigned to two conditions for pretreatment: (i) standard water supply (control) or (ii) solution of 200 μmol⋅L^-1^ melatonin in water (treatment). The pretreatment was conducted from September 14 to October 11 in an open experimental field. During this period, the seedlings were treated with melatonin or water every 7 days by root irrigation (20 mL per pot). After the fifth irrigation, the plants were sampled at day 0 (October 12), day 15 (October 5), day 30 (November 11), and day 45 (November 26), between 10:00 and 11:00 h, by removing the fifth to ninth leaves upward along the stem from five trees per treatment. Each treatment contained 45 pots (1 seedling per pot). The samples were quickly frozen after collection and stored in a cryogenic refrigerator at -80°C for subsequent index determination. All reactions were performed by using the leaf mixture of five kiwifruit seedlings with three technical and three biological replicates.

### Measurement of Chlorophyll, Malondialdehyde (MDA), and Hydrogen Peroxide (H_2_O_2_) Concentration

The chlorophyll concentration was measured as described by Arnon (1949); chlorophyll was extracted with 80% acetone, and the concentrations of chlorophyll a and b were determined with a UV-1800 system (Shimadzu, Kyoto, Japan). MDA content was measured according to the thiobarbituric acid method (Hodges et al., 1999). Briefly, 0.3 g leaves was ground as homogenate using 5 ml cold 5% (w/v) trichloroacetic acid solution (TCA) and centrifuged at 10000 *g* for 10 min at 4°C. Then, 2 ml supernatant and 0.67% (w/v) thiobarbituric acid (TBA) were mixed, sealed and heated for 10 min in boiling water bath. Following boiling water bath, the absorbance of supernatant was measured at 450, 532, 600 nm after cooling. Determination of H_2_O_2_ concentration was based on the method of Lin et al. (1988). In brief, 0.3 g leaves was ground as homogenate with 5 ml cold acetone and centrifuged at 10000 *g* for 10 min for 4°C. Then, 1 ml supernatant was added to 0.1 ml 5% (w/v) titanium sulfate and 0.2 mL concentrated ammonia and the mixture was mixed. The mixture was centrifuged at 4000 *g* for 10 min for 4°C. After centrifuging, the precipitation was saved and washed for 3–5 times with acetone until the plant pigment was removed. Finally, the precipitation was dissolved with 5 mL 2 M concentrated sulfuric acid and fix the volume of distilled water to 10 ml. The absorbance was determined at 415 nm. The content of H_2_O_2_ was based on a standard curve generated with known H_2_O_2_ concentrations.

### Determination of Osmotic Substance Content and Activity of Antioxidant Enzymes

Content of soluble protein and soluble sugar, as well as the activities of POD, SOD, CAT were determined using the method of Wang and Huang (2015) The content of soluble sugar was determined using anthrone colorimetry method. 0.2 g leaves and deionized water were extracted in boiling water bath for 30 min for twice. The extract was added to ethyl acetate containing anthrone and concentrated sulfuric acid and the mixture was mixed. Then the mixture was induced in boiling water bath about 1 min and the absorbance was determined at 630 nm. Soluble protein content was measured by Coomassie brilliant blue G-250 method. In brief, 0.3 g of sample leaves was ground as homogenate with 50 mM cold potassium phosphate buffer (PBS) (pH 7.8). The homogenate was centrifuged at 10000 *g* for 10 min at 4°C and the supernatant was saved for further analysis. The supernatant was added to coomassie brilliant blue G250 solution (dissolved in 90% ethanol and 85% (w/v) phosphoric acid) and the mixture was mixed. The absorbance was determined at 595 nm.

For the extraction of crude enzyme solution, 0.3g leaves was ground as homogenate in 8 mL cold 50 mM PBS (pH 7.8) containing 1% (w/v) polyvinyl pyrrolidone (PVP), 2 mM dithiothreitol (DTT) and 0.1 mM ethylenediaminetetraacetic acid (EDTA). Homogenates were then centrifuged at 10000 *g* at 4°C for 10 min and the supernatant was saved for further enzyme activity measurement. The guaiacol colorimetry was used for the measurement of POD activity. POD activity was measured in a reaction mixture containing 50 mM PBS (pH 5.5), guaiacol, 30% (v/v) H_2_O_2_, and the enzyme extract. The absorbance change in 470 nm was monitored and the result was expressed as U.g^-1^ (absorbance decrease of 0.01 per minute is 1 U at 470 nm). SOD activity was determined based on photochemical reduction of nitro blue tetrazolium (NBT) and assayed by monitoring the absorbance at 560 nm. The result was expressed as U.g^-1^ (reaction mixture absorbance of 1 g kiwifruit leaf at 470 nm decrease of 0.01 per minute is 1 U). The activity of CAT was determined by monitoring the decline in 240 nm and the result was expressed as U.g^-1^ (reaction mixture absorbance of 1 g kiwifruit leaf at 240 nm decrease of 0.1 per minute is 1 U). The reaction mixture containing 200 mM PBS (pH 7.8), 100 mM H_2_O_2_, and the enzyme extract.

### Determination of AsA and GSH Content

AsA content was determined by the Fe^3+^ reduction method (Kampfenkel et al., 1995). In brief, 0.5 g of sample leaves was ground as homogenate using 5 mL cold 5% (w/v) TCA. The homogenate was centrifuged at 10000 *g* for 10 min at 4°C and the supernatant was saved for further analysis. For the measurement of total ascorbate (T-AsA), 0.2 mL supernatant was incubated in 200 mM PBS (pH 7.4) and 6 mM DTT mixture at 42°C for 20 min in a water bath. After incubation, 0.2 ml 0.4% (w/v) *N*-ethylmaleimide (NEM) was added to remove excess DTT. Then 1 mL 10% (w/v) TCA, 0.8 mL 42% (w/v) o-phosphoric acid, 0.8 mL 2% (w/v) 2, 2′-dipyridyl in 70% ethanol, and 0.4 mL 3% (w/v) FeCl_3_ were added to the reaction mixture. The reaction was incubated at 42°C for 50 min in a water bath. AsA content was measured by using the similar method described above except DTT and NEM were substituted with 0.4 mL deionized water. The absorbance of AsA and T-AsA reaction mixture were determined at 525 nm by an ultraviolet spectrophotometer. T-AsA and AsA contents were determined based on a standard curve generated with known AsA concentrations. Dehydroascorbate (DHA) was defined as the difference between T-AsA and AsA.

The measurement of GSH content was based on the method of Griffith (1980); Briefly, 0.5 g of leaf tissue was ground as homogenate using 5 mL cold 7% (w/v) sulfosalicylic acid. The homogenate was centrifuged at 10000 *g* for 10 min at 4°C and the supernatant was saved for further analysis. For total glutathione (T-GSH) content analysis, 0.1 mL supernatant was mixed with 2.0 mL 200 mM PBS (pH 7.0), 0.3 mL 3 mM dithiobis-2-nitrobenzoicacid (DTNB), 0.3 mL 0.5 mM NADPH (include 7 mM EDTA). The reaction was initiated by adding five units of GR and incubated at 27°C for 30 min in a water bath. Glutathione disulfide (GSSG) content was analyzed in a same method as above, except for that the volume of PBS was substituted with 1.7 ml and the 0.1 mL supernatant was first incubated with 0.3 mL 2-vinylpyridine (2-VP) at 27°C for 1 h to derivatize GSH. The absorbance was measured at 412 nm for the reaction mixture of T-GSH and GSSG. T-GSH and GSSG contents were determined based on a standard curve generated with known GSH concentrations. GSH content was the difference between T-GSH and GSSS.

### Determination of the Content of Phenolic Compounds and Antioxidant Capacity

The methods of determining total phenolics (TPC), flavonoids (TFC), flavanols (TFAC), and anthocyanins (TMAC) were described by Wang (2015). In brief, 0.2 g of leaf tissue was ground as homogenate with cold 70% (v/v) methanol containing 2% (v/v) formic acid and 28% (v/v) ethanol. The homogenate was ultrasonically extracted for 30 min and shake at 250 rpm for 2 h at 30°C. Then the homogenate was centrifuged at 10000 *g* for 10 min at 4°C and the supernatant was filtrated by 0.45 μm filter membrane for further analysis. TPC was determined by folin-ciocaleu method, and the absorbance was measured using an ultraviolet spectrophotometer at 765 nm by using gallic acid as standard; the result was represented by gallic acid equivalency. The absorption of TFC was measured at 510 nm, and expressed as rutin equivalents. TFAC was determined by p-DMACA method; absorbance was determined at 640 nm, and the result was expressed as catechin equivalents. The pH differential was used to measure TMAC. The fruit extract was diluted to pH 1.0 and 4.5 using a buffer solution and the absorbance value at 510 and 700 nm was measured at each pH. The total anthocyanin content was calculated as the difference between them. The methods of Du (2009) were applied to measure free radical scavenging ability, including DPPH, ABTS, and FRAP methods. The results of these three methods were expressed as trolox equal antioxidant capacity.

### Quantitative Polymerase Chain Reaction (PCR) Analysis

Quantitative PCR (qPCR) was used to analyze the transcript levels of genes involved in the synthesis of flavonoids and AsA-GSH cycle in naturally senescencing seedlings. The Primer3 INPUT^1^ was used to design primers (**Table 1**). OmniPlant RNA Kit (DNase I) (CoWin, China) was used to extract total RNA, according to the manufacturer’s instructions. One microgram total RNA was used to synthesize the first strand cDNA using PrimeScript^TM^ RT reagent Kit with gDNA Eraser (Perfect Real Time) (Takara, Japan). The synthesis was performed according to the instructions of the manufacturer. A qPCR was performed with a SYBR Premix Ex Taq^TM^ II Kit (TaKaRa, Japan) on Real-Time System (CFX96, Bio-Rad, ıHercules, CA, United States). The reaction mixture (20 μL) contained 1.5 μL cDNA (100 times dilution), 0.8 μL each primer (10 μmol L^-1^), 10 μL 2× SYBR Premix Ex Taq^TM^ II (Tli RNaseH Plus) (TaKaRa, Japan), and 6.9 μL ddH_2_O. The reaction conditions were as follows: 95°C for 30 s, followed by 40 cycles of 95°C for 10 s, and 58°C for 30 s. As an internal control, Actin (Atkinson et al., 2009) (**Table 1**) was used to normalize the relative expression levels of the genes studied. Three PCR replicates were conducted per sample and the 2^-ΔΔ*C*^_T_ method was applied to calculate the relative expression levels. Total RNA were extracted in the sample leaves with three biological replicates, three technical replicates were conducted when qPCR was performed.

**Table 1:** The primer sequence.

Gene name	Primer	sequence	Gene ID
kiwifruit *ACTIN*	F	TGCATGAGCGATCAAGTTTCAAG	
	R	TGTCCCATGTCTGGTTGATGACT	
*CAB*	F	CGCGCACACATATACCAATC	Achn252871
	R	GTTGAAGAGAGGCCAACAGC	
*APX*	F	TCTTCACAGCTTTCGCATCT	Achn187071
	R	AGCATTAGCACGGTATCCTT	
*GR*	F	CCTAATGAAGTCGAGGTGAC	Achn214751
	R	GCCAGTTGCGATGAGTATGT	
*MDAR*	F	GACTACCTGCCGTTCTTCTA	Achn297231
	R	CTATCTCGCCTACACCATCT	
*DHAR*	F	AAAACATCTCCCTTACGACA	Achn278191
	R	CTTTACCTTCTGGGCTTATT	
*PAL*	F	CCTCTTGCCGAAAGAAGTTG	Achn060261
	R	CCTGTTTGGAATTGCTGGAT	
*C4H*	F	CCCCTTCTAGTCCCTCACA	Achn214151
	R	GCCACCAAGCGTTTACCA	
*CHS*	F	GCCAAAGACCTAGCAGAG	Achn238171
	R	AGAGCAGACGACCAATACC	
*FNS*	F	ATGGCCATTTTCTGAGCAAC	Achn075731
	R	TGCTGCAATTTGAGTTCACC	
*F3H*	F	TGCCCTCAACCAGACCTCA	Achn201561
	R	ATTCTTCCCACCGTCCCT	
*LAR*	F	TTTCCAGCCTCAATCCAATC	Achn166621
	R	CGAGCTTCTTCTCCCACAAC	
*ANR*	F	TCGATACCTTTGCTGTGCTG	Achn030761
	R	ATCAACTTGGCTTTGGATGG	
*UFGT*	F	CGCAAGGATGATGAGTCAGA	Achn385311
	R	GCAACCTCACTGCCTTCTTC	


### Statistical Analysis

Excel 2010 was used for data processing. The data were plotted using SigmaPlot 12.5 (Systat, Santa Clara, CA, United States). Analysis of variance was performed by the statistical program SPSS 22.0 (SPSS, Inc., Chicago, IL, United States). Significant differences were detected by Duncan’s multiple range tests at the 0.05 level.

## Result

### Effect of Melatonin Application on the Physiological State of the Leaves of Kiwifruit During Senescence

**FIGURE 1:**
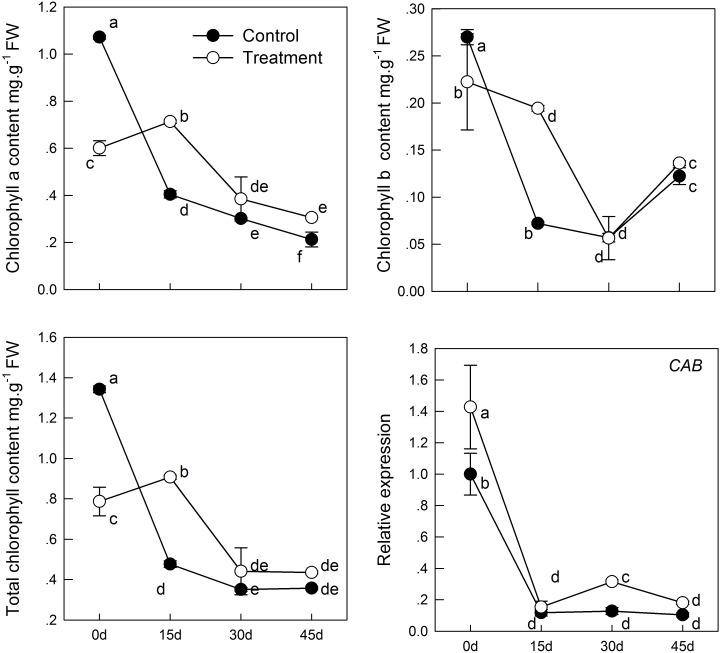
The effect of melatonin on leaves chlorophyll content and *CAB* gene analyzed by qPCR during kiwifruit senescence. Data are show as means ± SE (*n* = 9), different letters indicate significant differences at *p* < 0.05 level.

To determine whether supplemental melatonin can delay the senescence, we detected the chlorophyll content, soluble sugar, and soluble protein of kiwifruit leaves in different treatments. We found that melatonin pretreatment decreased chlorophyll degradation (**Figure 1**). During natural senescence, the concentrations both chlorophyll a and b of the melatonin-treated group were higher than that of control group after day 0; particularly on day 15, total chlorophyll concentration of the melatonin-treated group was 1.9 times of that of the control. However, in later senescence processes, the efficacy of melatonin application reduced gradually, indicated by the lack of any significant difference between the two groups. CAB was responsible for encoding chlorophyll a/b-binding protein. The expression of *CAB* was down-regulated during senescence, but the *CAB* expression in samples pretreated with melatonin was slightly higher than that in the control group. However, there was no significant difference between day 15 and 45 in the control group, and the result appeared consistent with the change in chlorophyll content.

**FIGURE 2:**
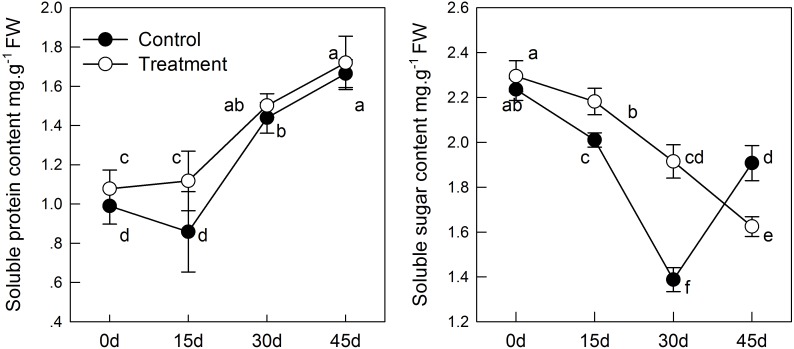
The effect of melatonin on leaves physiological state of kiwifruit during senescence. Data are show as means ± SE (*n* = 9), different letters indicate significant differences at *p* < 0.05 level.

With aging, the content of soluble sugar in the leaf samples decreased continuously (**Figure 2**), which may induced by the decrease in total chlorophyll concentration, thereby causing reduction of carbohydrates synthesis by photosynthesis. However, melatonin treatment delayed the decrease in soluble sugar content. The soluble sugar content in the sampled leaves treated with melatonin was significantly higher in the treatment than in the group before 30 days. Furthermore, melatonin treatment promoted the content of soluble protein (**Figure 2**), which was 1.3 times as high in the melatonin-treated group as that in the control group on day 15. However, there was no significant difference between the treatment and control groups after 15 days.

### Effects of Exogenous Melatonin Application on Antioxidant System of Kiwifruit Leaves During Natural Senescence

**FIGURE 3:**
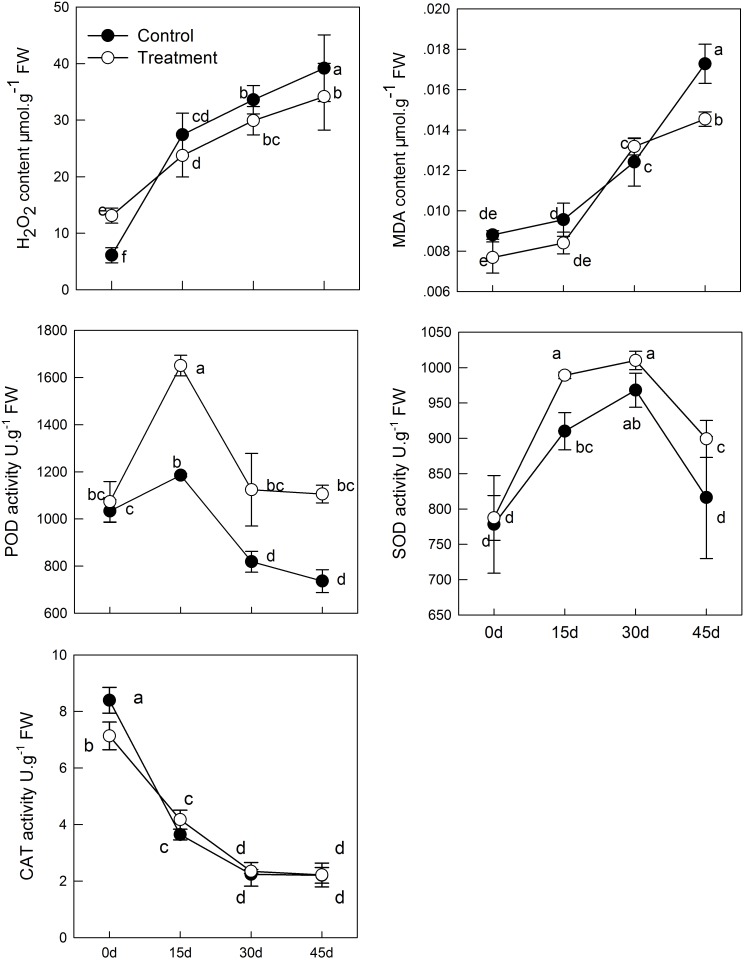
The effects of melatonin on antioxidant system of kiwifruit during senescence; Data are show as means ± SE (*n* = 9), different letters indicate significant differences at *p* < 0.05 level.

The MDA content indicates lipid peroxidation in plant cell membranes. In the senescence process, melatonin treatment can alleviate the membrane peroxidation process induced by aging; but the difference between the treatment and control groups was not significant before 30 days (**Figure 3**). Similar results as those mentioned above were obtained when monitoring H_2_O_2_ levels. H_2_O_2_ concentration increased with aging, whereas H_2_O_2_ was reduced in tissues sampled from pretreated seedlings. On day 45, the content of H_2_O_2_ in the melatonin-treated group was significantly lower than that in the control group (**Figure 3**). These results indicated that melatonin might partly control the ROS concentration in leaves and alleviate membrane lipid oxidation.

In the process of natural senescence, POD activity in the melatonin-treated group was significantly higher than that in the control group (**Figure 3**). When the melatonin- pretreated seedlings were at day 45, the activity of POD was higher than that in the control seedlings. CAT activity in pretreated seedlings was slightly higher than that in the control group, but the difference was not significant. Likewise, melatonin strongly activated the activity of SOD of treatment group; SOD had maximum activity on day 30. The results were consistent with the changes of H_2_O_2_ content, which indicated that melatonin could promote the activity of antioxidant enzymes and enhance the antioxidant ability of kiwifruit.

### Effect of Melatonin Application on the AsA-GSH Cycle in Leaves of Kiwifruit During Senescence

**FIGURE 4:**
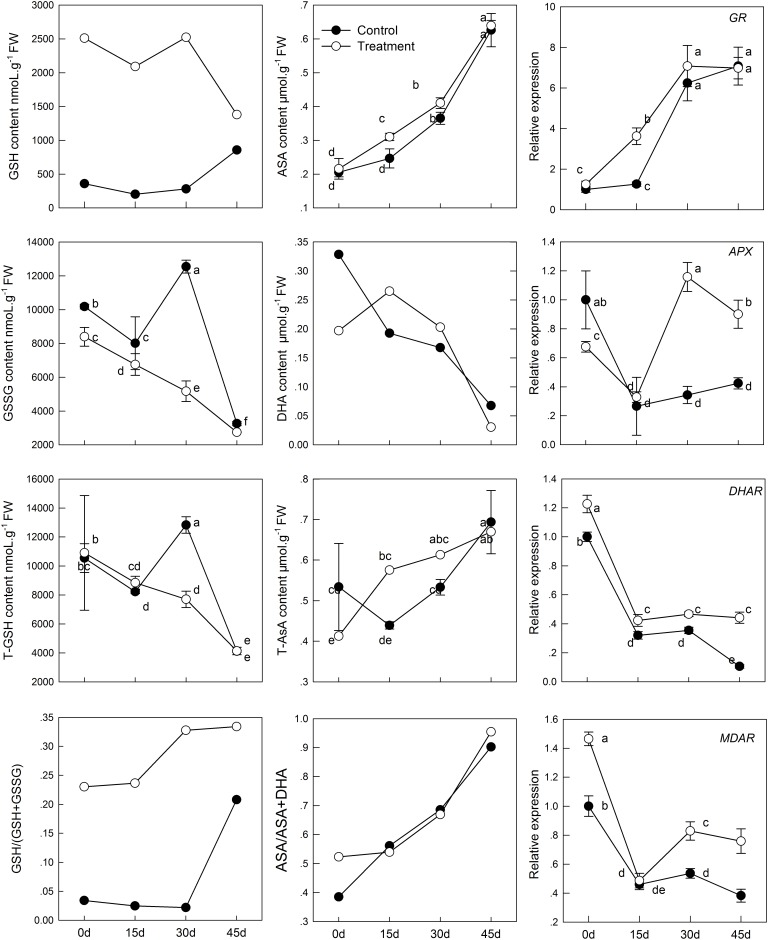
Effects of exogenous melatonin on AsA-GSH cycle and genes that analyzed by qPCR involved in AsA-GSH cycle during kiwifruit senescence; Data are show as means ± SE (*n* = 9), different letters indicate significant differences at *p* < 0.05 level.

The intracellular redox state conversion of ascorbic acid and glutathione plays an important role in resistance of plants to stress and senescence. In the process of senescence, T-GSH and GSSG content decreased constantly; simultaneously, melatonin pretreatment increased GSH content and decreased GSSG content (**Figure 4**). However, the T-GSH content in control was significantly higher than in the melatonin-treated group on day 30, which was the result of GSSG accumulation. Similarly, the content of both T-AsA and AsA were promoted in tissues sampled from pretreated seedlings. The resultant DHA produced in the melatonin-treated group was higher than that in the control on day 15 and 30 owing to continuous oxidation of AsA. Meanwhile, a higher rate of AsA/(AsA + DHA) and GSH/(GSH + GSSG) was found in the melatonin-treated samples with aging than in the untreated samples.

The transcript levels of four genes related to AsA-GSH cycle (*GR*, *APX*, *DHAR*, and *MDAR*) in leaves of seedlings were found to be significantly activated by pretreatment with melatonin (**Figure 4**). The *GR* expression level of melatonin-treated group was slightly higher than that of the control group, but exhibited no significant difference, except at day 15. *APX* expression was lower in the melatonin-treated group than in the control group in the earlier period, but it was up-regulated by melatonin after day 15. The maximum expression was 3.37 times as that of the control group on day 30. The expression of *DHAR* and *MDAR* was down-regulated. However, it was markedly increased by melatonin treatment. On day 45, *DHAR* and *MDAR* of the melatonin-treated group was 4.18 and 1.98 fold higher than that of the control group, respectively.

### Effect of Melatonin Application on Flavonoid Biosynthesis in Leaves of Kiwifruit During Senescence

**FIGURE 5:**
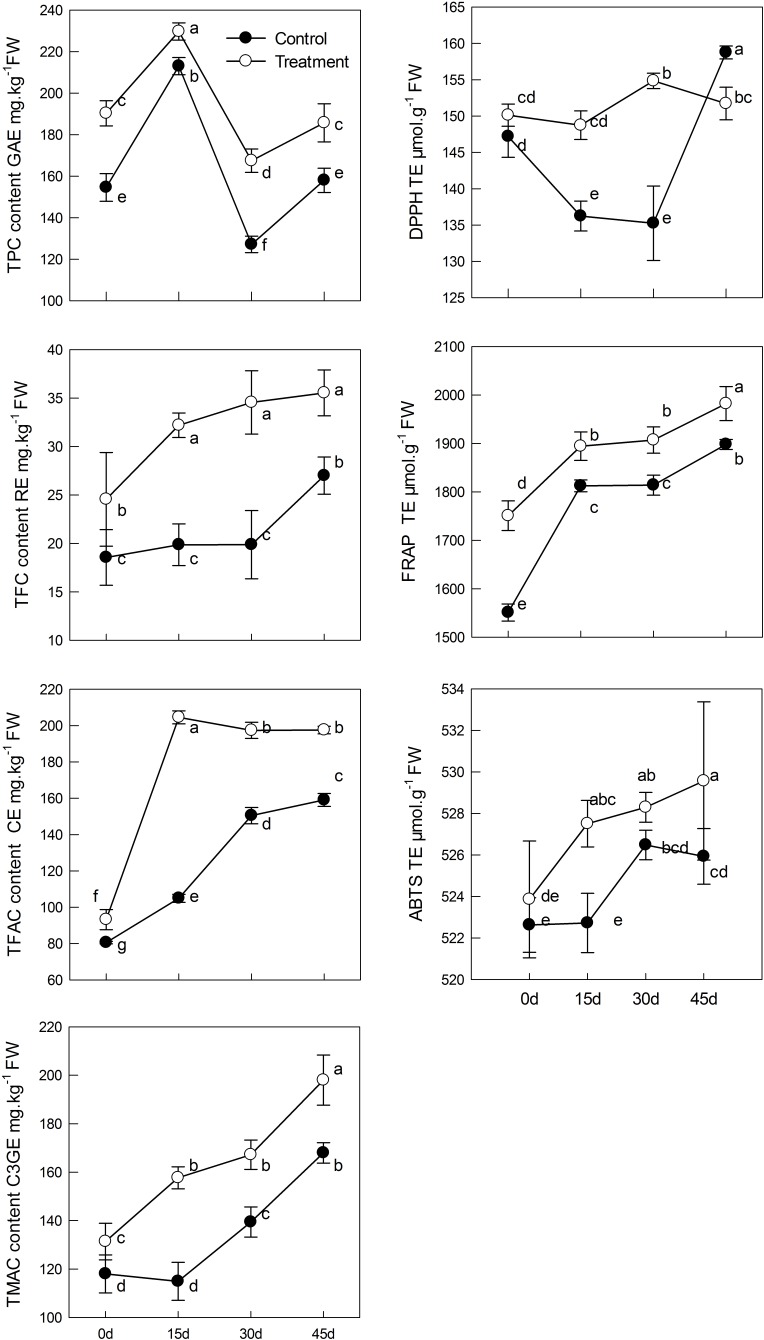
The effects of melatonin on phenolic compounds and antioxidant capacity determined by DPPH, ABTS, and FRAP assays of kiwifruit during senescence; Data are show as means ± SE (*n* = 9), different letters indicate significant differences at *p* < 0.05 level.

Phenolic compounds are one of foremost and widely distributed secondary metabolites in plants, which can enhance plant stress resistance and natural antioxidant ability. We found that phenolic compound content in the course of natural senescence was significantly improved by melatonin application, as compared with that in the control group (**Figure 5**). In pretreated seedlings, The TPC reached the maximum on day 15 (increased by 21%); thereafter TPC began to decrease gradually, and at day 45, there was no significant difference from day 0. However, TFC constantly increased to levels that were 1.3 times higher in the melatonin-treated group that in the control group at day 45. Moreover, TFAC began to stabilize after reaching a maximum value on day 15. In accordance with TFC, TMAC in the melatonin-treated group was markedly higher than that in the control group. It increased 50.7% from day 0 to day 45. The anti-oxidation ability in sample leaves was also promoted by melatonin pretreatment. Both the DPPH, FRAP, ABTS, and antioxidant capacity of the melatonin-treated group was significantly higher than that in the control group, which demonstrated that melatonin can directly or indirectly regulate the antioxidant ability of plants as an antioxidant.

**FIGURE 6:**
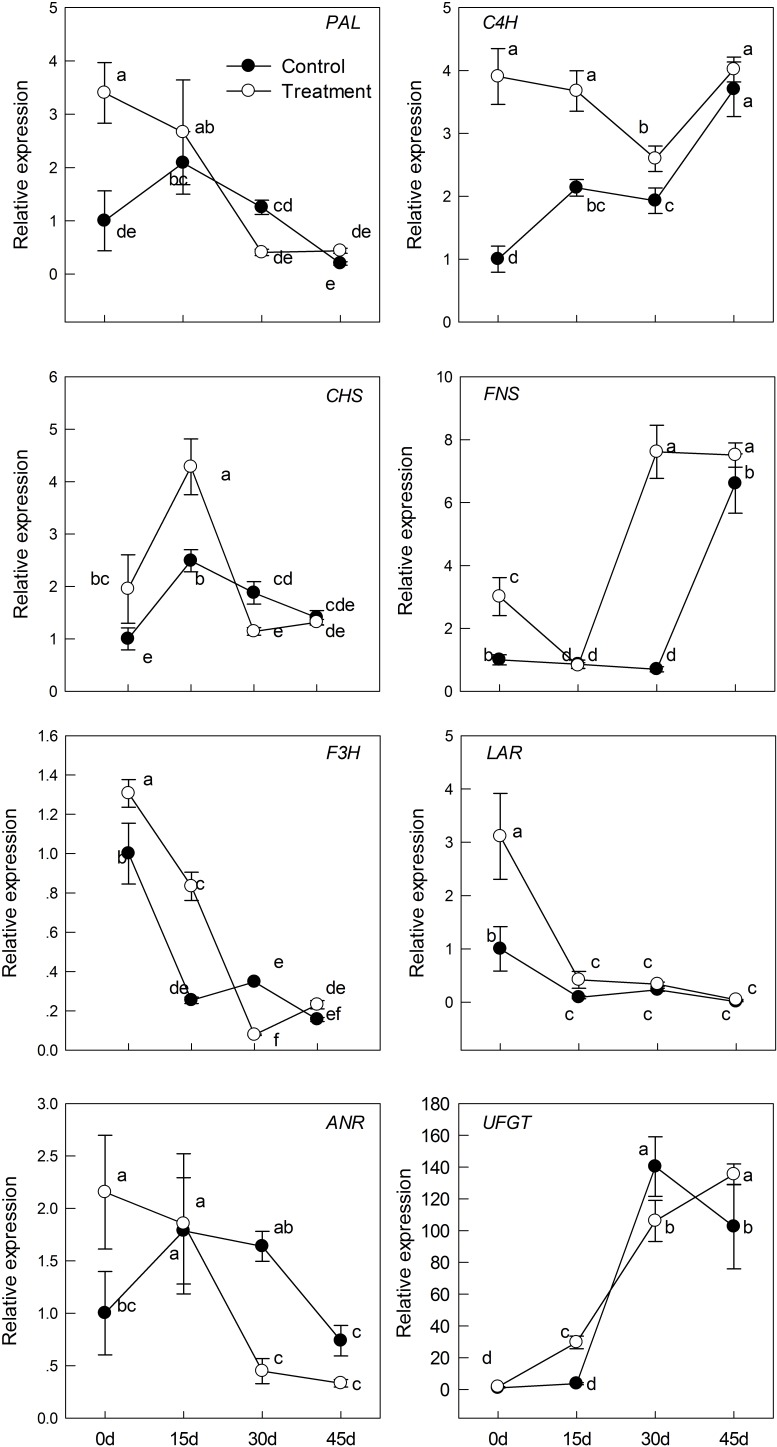
The effects of melatonin on flavonoids biosynthesis genes that analyzed by qPCR during kiwifruit senescence; Data are show as means ± SE (*n* = 9), different letters indicate significant differences at *p* < 0.05 level.

For further dissecting melatonin modulation of controlling flavonoids metabolism, we measured the mRNA levels of *PAL*, *C4H*, *CHS*, *FNS*, *F3H*, *LAR*, *ANR*, and *UFGT* by real-time quantitative PCR, both are the crucial genes related flavonoids metabolism (**Figure 6**). PAL and C4H are all rate-limiting enzymes in the first two steps of the flavonoid synthesis pathway; we observed that transcript levels of two genes induced by melatonin were significantly higher in the melatonin-treated group than in the control group. Similarly, the expression levels of TMAC biosynthesis structural genes (*CHS*, *FNS*, *F3H*, and *UFGT*) were markedly higher in melatonin pretreatment than in the control, but the expression level of *CHS* and *F3H* was down-regulated. There was no significant difference in *CHS* and *F3H* expression between the melatonin-treated group and the control group after day 30. Moreover, in the melatonin-treated group, *FNS* expression reached the maximum (approximately 10.87-fold of that of the control group) at 30 days. Melatonin also increased the transcript level of the proanthocyanidin biosynthesis genes, *LAR* and *ANR*, but it was significant only before day 15; thereafter, the effect of melatonin gradually decreased with the process of senescence. There was roughly no difference among the control and melatonin-treated groups. Various expression patterns were observed among the flavonoid biosynthesis pathway genes, but in all, melatonin played a catalytic role in the expression. This result also coincided with the significant increase of TPC, TFC, TFAC, and TMAC (**Figure 5**).

## Discussion

The most obvious indicators of plant senescence are the change in leaf color, from green to yellow, and leaves falling off, while chloroplast disintegration and chlorophyll degradation appear at the cellular level. Variation in leaf chlorophyll concentration is an important indicator of leaf physiological activity, making it a valuable, intuitive method to determine the effect of an exogenous substance on leaf senescence. Present results showed that melatonin clearly has a role in delaying leaf senescence, as observed by the maintenance of chlorophyll content (**Figure 1**) in the treatment group. One key gene involved in chlorophyll biosynthesis, *CAB*, which encodes the chlorophyll a/b-binding protein, is down-regulated in all groups; however, supplement of melatonin promoted transcript level of *CAB* compared to control group. In previous study, short-term and long-term application of exogenous melatonin to apple trees resulted in more chlorophyll content and delayed leaf senescence (Wang et al., 2013b,c, 2016). Besides, the protective effect of melatonin on chlorophyll was also verified in barley (Arnao and Hernandez-Ruiz, 2009) and photosynthetic green alga (*Ulva* sp.) (Tal et al., 2011). All of these findings demonstrated that melatonin is a positive regulator of plant aging, and that exogenous applications delay senescence.

Leaf senescence is accompanied by many physiological processes, such as the accumulation of H_2_O_2_, increased membrane permeability, and release of protein and other contents. Our findings indicated that exogenous melatonin delayed senescence: seedlings pretreated with melatonin had lower H_2_O_2_ and MDA concentration. H_2_O_2_ is an important ROS in plants and is accumulated under stress. Accumulation of ROS can accelerate membrane liposylation of cells and produce the toxic substance, MDA, thus increasing membrane permeability of plants and damaging the integrity of the membrane structure. In the experiment, H_2_O_2_ and MDA content continuously increased with the aging process, but the content in pretreated seedlings was lower than that in CK. This result can be partly attributed to the fact that melatonin is a free radical scavenger and broad-spectrum antioxidant, and can remove H_2_O_2_ involved in the aging process directly and in time, which is helpful to maintain homeostasis in cells (Tan et al., 2007); this was similarly seen in apples (Wang P. et al., 2012), cucumber (Marta et al., 2016), and peach (Gao et al., 2016). SOD, POD, and CAT are three important protective enzymes in plant growth and defense. O^2-^ in a plant can be reduced to H_2_O_2_ by SOD, and then H_2_O_2_ will be completely decomposed as H_2_O and O_2_ by POD and CAT; this will alleviate damage to the membrane system (Tao et al., 2008). We found that, during aging, the difference in SOD and POD activity was extremely significant between the melatonin-pretreated group and the control group. CAT activity was continuously decreased with the aging process, and no difference between control and treatment, but also higher than control slightly. This has been demonstrated in experiments with the *Malus* plant where melatonin directly scavenges H_2_O_2_ and enhances the activities of the antioxidant enzyme to detoxify H_2_O_2_ in plants under salt (Jiang et al., 2016; Li et al., 2017a), drought (Wang et al., 2013c), and alkaline (Gong et al., 2017) stress. In watermelon, local application of melatonin activates these enzymes to remove excess ROS and make plants tolerant to cold stress (Li et al., 2017b). These finding suggested that melatonin has a significant effect on delaying kiwifruit senescence.

The soluble sugar content decreased with age, but the soluble protein content showed the opposite trend. The soluble sugar and soluble protein content were higher in pretreated seedlings than in control seedlings (**Figure 2**), which is consistent the results of the study by Wang et al. who suggested that soluble sugar and soluble protein content in apples under salt stress changed in response to stress (Wang et al., 2013a). Likewise, melatonin treatment increased the content of soluble sugar and slowed its degradation during senescence, suggesting the potential function of melatonin in increasing crop yield. Overall, chlorophyll content in the melatonin-treated group was higher than in the control group. Chlorophyll converts energy (photons) into its own carbohydrates through photosynthesis. The up-regulation expression of *CAB* and higher chlorophyll content were beneficial for the accumulation of soluble sugars and soluble proteins. Both of which can balance the cell metabolism during leaf senescence, thus maintaining cellular homeostasis.

The AsA-GSH cycle is an important way for eliminating free radicals in plants. It has been reported that the production of GSH and AsA is induced by melatonin under drought stress and is associated with low H_2_O_2_ content in tomato (Liu et al., 2015). Under stress, AsA and GSH in the cells were oxidized, and there was a similar transformation during leaf senescence (Borraccino et al., 1994). Wang et al. also discovered that delayed senescence of apple leaves by exogenous melatonin treatment regulates the AsA-GSH cycle (Wang P. et al., 2012). In the present study, we observed T-AsA and AsA content in the melatonin-pretreated leaves were significantly higher than those in the untreated controls. Meanwhile, down-regulation of *MDAR* and *DHAR* expression were found, but melatonin markedly improved the transcript level of *MDAR* and *DHAR*. It indicated that higher expression of *MDAR* and *DHAR* guaranteed higher T-AsA and AsA content. In this experiment, melatonin also maintained higher GSH content and total GSH content in senescent leaves. The proportion of GSH in T-GSH was also higher in the melatonin-treated group than that in control. Up-regulation of *GR* gene was observed in melatonin-treated seedlings in this study, which corresponded with the decreased GSSG content. APX can utilize AsA to reduce H_2_O_2_ to H_2_O and produce DHA and MDA, the expression of which was promoted by melatonin as well. The decreased H_2_O_2_ content owing to treatment with melatonin may be related to the high expression of *APX* and the higher AsA and GSH content. Our findings suggest that melatonin plays a vital role in the biosynthesis of GSH and AsA and highlights the role of AsA-GSH and melatonin in ROS balance.

Phenolic compounds are the secondary metabolites with antioxidant activity in plants, and they may act as the second line of defense to participate in the scavenging process of ROS (Fini et al., 2012; Wang, 2015). In tomato, the application of exogenous melatonin increased the production of eight proteins involved in anthocyanin accumulation during fruit ripening; therefore, the total anthocyanin content was increased after melatonin treatment.(Sun et al., 2016). In the present study, our treatment evidently improved the accumulation of phenolic compounds. Moreover, we measured the mRNA levels of *PAL*, *C4H*, *CHS*, *FNS*, *F3H*, *LAR*, *ANR*, and *UFGT* by qPCR and found that melatonin was also involved in the regulation of those genes. PAL and C4H are rate-limiting enzymes in the first two steps of the flavonoid synthesis pathway. *CHS*, *FNS*, *F3H*, and *UFGT* are the structural genes of anthocyanin biosynthesis, and *LAR* and *ANR* are related to proanthocyanidins biosynthesis. We observed the genes *PAL*, *C4H*, *CHS*, *F3H*, *LAR*, *ANR*, and *UFGT* exhibited higher expression level in treatment group than control before 15 days (**Figure 6**). Moreover, the treatment content of TPC, TFC, TFAC, and TMAC reached the maximum or stable value and also significantly higher than control group on 15 days (**Figure 5**). Consequently, the accumulation of phenolic compounds attributed to the higher expression level of those genes before 15 days. While the gene *FNS* presented higher transcript level after 15 days, the result indicated the reason why TPC content constantly increased. Additionally, the expression level of genes *PAL*, *CHS*, *F3H*, and *UFGT* in treatment group presented no difference or lower than control after 15 days, the TPC and TFAC content decreased accordingly. The TMAC content reached the maximum on 45 days, which phenomenon may explicate by the interaction of gene *LAR* and *ANR*. Although these genes showed different expression patterns with aging, melatonin treatment improved in the melatonin-treated group than in the control group. Overall, our results indicated that the presence of direct or indirect cross-talk between melatonin and flavonoids for playing important roles in delaying leaf senescence. DPPH, ABTS, and FRAP are three commonly used methods for the determination of antioxidant activity, which can comprehensively evaluate the antioxidant capacity of plants (Wang X.Y. et al., 2012). We observed that antioxidant activity was enhanced in melatonin-pretreated leaves, which was in accordance with the trends of increased content of phenolic compounds.

**FIGURE 7:**
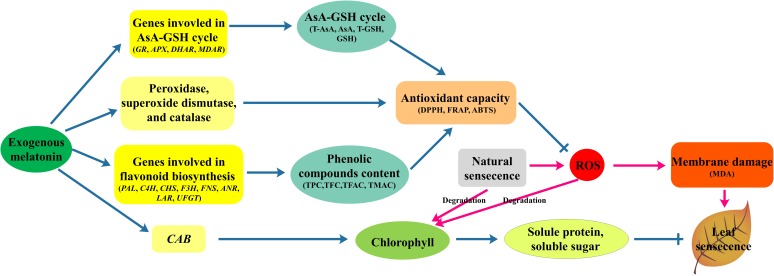
The mechanisms of melatonin regulation leaf natural-senescence derived from results involving antioxidant metabolism and biosynthesis of flavonoids.

In summary, 200 μM melatonin treatment significantly delayed natural-senescence of kiwifruit leaf. The exogenous application of melatonin effectively promoted the transcription of *CAB*, slowed chlorophyll degradation, resulted more soluble protein and soluble sugar accumulation and maintained the balance of cell metabolism. Supplementary of melatonin also promoted the activity of SOD, POD, and CAT. Melatonin may influence both antioxidant enzyme activity as an antioxidant and cellular mRNA levels for these enzymes (Rodriguez et al., 2004). In addition, the expression of genes involved in AsA-GSH cycle significantly promoted by melatonin-pretreatment, as a result the content of antioxidant substances (AsA, GSH) strongly increased. Moreover, a large amount of flavonoids accumulated in seedlings pretreated with melatonin, and transcript levels of eight genes involved in flavonoid synthesis were increased in response to melatonin application. Owing to the higher antioxidant enzymes activity and more antioxidant substances in leaves melatonin-pretreated, the treatment group had higher antioxidant ability to scavenging ROS, increase the stability of cell membrane as manifested by lower MDA content compared to control. Our results demonstrated that the mechanisms of melatonin delayed leaf natural-senescence through regulating antioxidant metabolism and biosynthesis of flavonoids, as depicted in **Figure 7**. The finding can provide clear proof that melatonin plays a key roles in delaying leaf senescence.

## Author Contributions

YS carried out the experiments with the help of NX, QW, and ZL. HX and DL provided all critical intellectual inputs into the study design. YS, DL, and ZN collected the experimental data and drafted the manuscript. QD, LL, XL, HX, and JW provided suggestions for the revision of the manuscript. All authors read and approved the final manuscript.

## Conflict of Interest Statement

The authors declare that the research was conducted in the absence of any commercial or financial relationships that could be construed as a potential conflict of interest.
